# Functional connectivity in the first year of life in infants at-risk for autism: a preliminary near-infrared spectroscopy study

**DOI:** 10.3389/fnhum.2013.00444

**Published:** 2013-08-06

**Authors:** Brandon Keehn, Jennifer B. Wagner, Helen Tager-Flusberg, Charles A. Nelson

**Affiliations:** ^1^Laboratories of Cognitive Neuroscience, Division of Developmental Medicine, Boston Children's HospitalBoston, MA, USA; ^2^Harvard Medical SchoolBoston, MA, USA; ^3^Department of Psychology, The College of Staten Island, The City University of New YorkStaten Island, NY, USA; ^4^Department of Psychology, Boston UniversityBoston, MA, USA

**Keywords:** autism, functional connectivity, near-infrared spectroscopy, endophenotype, infancy

## Abstract

**Background:** Autism spectrum disorder (ASD) has been called a “developmental disconnection syndrome,” however the majority of the research examining connectivity in ASD has been conducted exclusively with older children and adults. Yet, prior ASD research suggests that perturbations in neurodevelopmental trajectories begin as early as the first year of life. Prospective longitudinal studies of infants at risk for ASD may provide a window into the emergence of these aberrant patterns of connectivity. The current study employed functional connectivity near-infrared spectroscopy (NIRS) in order to examine the development of intra- and inter-hemispheric functional connectivity in high- and low-risk infants across the first year of life.

**Methods:** NIRS data were collected from 27 infants at high risk for autism (HRA) and 37 low-risk comparison (LRC) infants who contributed a total of 116 data sets at 3-, 6-, 9-, and 12-months. At each time point, HRA and LRC groups were matched on age, sex, head circumference, and Mullen Scales of Early Learning scores. Regions of interest (ROI) were selected from anterior and posterior locations of each hemisphere. The average time course for each ROI was calculated and correlations for each ROI pair were computed. Differences in functional connectivity were examined in a cross-sectional manner.

**Results:** At 3-months, HRA infants showed increased overall functional connectivity compared to LRC infants. This was the result of increased connectivity for intra- and inter-hemispheric ROI pairs. No significant differences were found between HRA and LRC infants at 6- and 9-months. However, by 12-months, HRA infants showed decreased connectivity relative to LRC infants.

**Conclusions:** Our preliminary results suggest that atypical functional connectivity may exist within the first year of life in HRA infants, providing support to the growing body of evidence that aberrant patterns of connectivity may be a potential endophenotype for ASD.

## Introduction

Autism spectrum disorder (ASD) is considered by many to be a “developmental disconnection syndrome” (Geschwind and Levitt, 2007), reflecting a shift in perspective from conceptualizing ASD as a disorder of region-specific dysfunction toward one associated with atypical neural circuitry (Belmonte et al., 2004; Muller, 2007; Wass, 2010). Despite being termed a developmental disconnection syndrome, the majority of the research examining anatomical and functional connectivity in ASD has focused on school-aged children, adolescents, and adults, with a small minority of imaging studies examining changes in connectivity across time. However, in ASD, perturbations in neurodevelopmental trajectories begin as early as the first year of life (e.g., Redcay and Courchesne, 2005), indicating that important neuropathological processes are operating in infancy if not earlier.

Prospective longitudinal studies of infants at high-risk for ASD (HRA; because they have an older sibling diagnosed with ASD) provide a window into the earliest manifestations of these aberrant patterns of neurofunctional and structural connectivity. Moreover, studies investigating siblings of individuals diagnosed with ASD also have potential to shed light on possible endophenotypes. Endophenotypes reflect characteristic behavioral or neurobiological features that are present in both affected individuals and their first-degree family members (Gottesman and Gould, 2003), and may lead to a more straightforward decomposition of complex genetic disorders, such as ASD. Both children with ASD and their siblings atypically evidence reductions in white matter connectivity (Barnea-Goraly et al., 2010). More recently, altered developmental trajectories of anatomical connectivity in high-risk infants later diagnosed with ASD were found (Wolff et al., 2012), indicating that atypical connectivity may represent an endophenotype or potential biomarker for ASD.

Much of the ASD anatomical and functional connectivity literature focusing on older individuals supports the underconnectivity theory of ASD originally put forward by Just et al. (2004, 2012). Findings from diffusion tensor imaging (DTI) studies, an imaging modality used to measure microstructural properties of white matter, have generally reported indices of reduced anatomical connectivity in school-aged children, adolescents, and adults with ASD (see Travers et al., 2012, for review). However, in contrast to DTI studies of older individuals with ASD, work with children with ASD as young as one-year-old has shown increased fractional anisotropy (FA) as compared to typically developing (TD) children (Ben Bashat et al., 2007; Weinstein et al., 2011). These results may be indicative of accelerated white matter development in ASD (although see Walker et al., 2012, for discussion of the difficulties interpreting DTI indices), and provide evidence that patterns of over- and under-connectivity may differ as a function of development.

Prior studies investigating connectivity using functional connectivity MRI (fcMRI), an analytical approach used to investigate inter-regional signal cross-correlations that reflect distributed functional networks, have reported both over- and under-connectivity in older individuals with ASD (which may be dependent on specific methodological decisions; see Muller et al., 2011). In the only study to examine functional connectivity in toddlers and younger children with ASD, Dinstein et al. (2011) reported reduced inter-hemispheric connectivity similar to findings from older individuals diagnosed with ASD. In typically developing infants, fcMRI analyses have shown that functional networks exist in neonates and mature gradually across the first years of life (see Smyser et al., 2011, for review). However, to date, no study has examined the development of functional brain networks in the first year of life in ASD.

Recently, functional connectivity has been investigated using near-infrared spectroscopy (fcNIRS) in typically developing infants (Homae et al., 2010, 2011; White et al., 2012) and adults (Mesquita et al., 2010; Zhang et al., 2010; Duan et al., 2012; Sasai et al., 2012). Near-infrared spectroscopy (NIRS) is a relatively new, non-invasive method used to measure concentrations of oxy- (oxy-Hb) and deoxy-hemoglobin (deoxy-Hb) in the cortex, and therefore provides an indirect measure of neuronal activity (similar to functional magnetic resonance imaging; fMRI) (see Gervain et al., 2011, for review). Unlike fMRI, NIRS does not require rigid head stabilization or that the infant be asleep (to avoid motion), making it a more suitable tool to study infant brain development. Furthermore, simultaneous NIRS-fcMRI studies have demonstrated that both methods produce similar functional networks (Duan et al., 2012; Sasai et al., 2012). The current study employed NIRS to examine functional connectivity in the first year of life in infants at high- and low-risk for ASD as they passively listened to linguistic stimuli (Gervain et al., 2008).

Although a failure of neurotypical development of functional brain networks is thought to characterize ASD, only a handful of studies have investigated early differences in connectivity. The current study addresses this gap in the literature by examining functional connectivity in infants at risk for autism as early as 3-months of age. Additionally, while our study is cross-sectional in nature, by investigating infants at risk for ASD we are able to examine *changes* in connectivity across the first year of life (before a reliable diagnosis of ASD can be made), and therefore provide insight into how atypical network organization may emerge in ASD. To our knowledge, this is the first study to examine functional connectivity in infants at risk for ASD. Specifically, the current study employed fcNIRS in order to examine the development of intra- and inter-hemispheric functional connectivity in high- and low-risk comparison (LRC) infants across the first year of life in order to determine if atypical connectivity represents an endophenotype in ASD.

## Materials and methods

### Participants

A total of 76 infants (*n* = 33 HRA; *n* = 43 LRC) completed visits at 3-, 6-, 9-, and/or 12-months of age. All infants had a minimum gestational age of 36 weeks, no history of prenatal or postnatal medical or neurological problems, and no known genetic disorders (e.g., fragile-X, tuberous sclerosis). Low-risk infants had a typically developing older sibling and no family history of autism or other neurodevelopmental disorders; infants at high-risk for ASD were defined by having at least one older full sibling with a diagnosis of Autistic disorder, Aspergers disorder, or Pervasive Developmental Disorder–Not Otherwise Specified. Community diagnosis of the older sibling with ASD was confirmed using the Social Communication Questionnaire (SCQ; Rutter et al., 2003). At 6- and 12-month visits, infants were administered the Mullen Scales of Early Learning (MSEL; Mullen, 1995) in order to obtain a measure of developmental functioning. Independent-samples *t*-tests and Fisher's Exact tests confirmed that, at 3-, 6-, 9-, and 12-month visits, HRA and LRC infants that contributed usable NIRS data did not differ significantly with regard to age, sex, head circumference, and at 6- and 12-months, did not differ on MSEL Early Learning Composite score (ELCS) (all *p* > 0.1) (see Table 1). Total attrition rates for the current study (26%) were similar to previous infant NIRS studies (~40%; see Lloyd-Fox et al., 2010, for review). At each visit time point, infants were excluded if they were unable to tolerate the NIRS hat, did not complete at least 14 blocks of the task, or did not have at least one usable channel in any region of interest (see Table 2 for more information). The final sample included a total of 64 infants (*n* = 27 HRA; *n* = 37 LRC) who contributed 116 data sets. Informed consent was obtained from all caregivers in accordance with the Boston Children's Hospital and Boston University Institutional Review Boards.

**Table 1:** **Participant information**.

	**HRA**				**LRC**			
	**3 months (*n* = 17)**	**6 months (*n* = 12)**	**9 months (*n* = 8)**	**12 months (*n* = 6)**	**3 months (*n* = 13)**	**6 months (*n* = 18)**	**9 months (*n* = 21)**	**12 months (*n* = 21)**
Age [days]	106 (11)	217 (15)	296 (17)	396 (13)	110 (12)	207 (17)	297 (17)	388 (13)
	91–121	193–242	272–329	382–416	94–135	186–241	276–336	364–422
Sex [males; females]	10; 7	5; 7	3; 5	2; 4	7; 6	8; 10	10; 11	11; 10
MSEL ELCS	n/a	91 (8)	n/a	105 (24)	n/a	93 (9)	n/a	103 (12)
		79–110		81–138		74–113		77–116
Blocks completed	27 (3)	26 (4)	27 (2)	27 (2)	27 (3)	28 (2)	27 (2)	27 (3)
	17–28	15–28	24–28	23–28	19–28	19–28	22–28	18–28
HC	0.38 (0.80)	0.78 (0.69)	1.0 (0.85)	1.1 (1.0)	0.58 (1.3)	0.99 (1.3)	0.99 (1.3)	0.84 (1.2)
	−0.9–1.8	0–2.3	−0.1–2.7	−0.5–2.3	−1.2–3.2	−0.6–3.4	−1.8–3.4	−1.3–3.2

Mean (SD); Range. Early Learning Composite Score (ELCS); Head circumference z-score (HC).

**Table 2:** **Attrition rates for entire sample of infants**.

	**HRA**				**LRC**				**Total**
	**3 months**	**6 months**	**9 months**	**12 months**	**3 months**	**6 months**	**9 months**	**12 months**	
Included	17 (94%)	12 (67%)	8 (62%)	6 (43%)	13 (100%)	18 (78%)	21 (78%)	21 (68%)	116 (74%)
Excluded: refused cap; fussed-out	0 (0%)	0 (0%)	1 (8%)	0 (0%)	0 (0%)	0 (0%)	1 (4%)	1 (3%)	3 (2%)
Excluded: <14 blocks administered	1 (6%)	4 (22%)	2 (15%)	5 (36%)	0 (0%)	1 (4%)	1 (4%)	2 (6%)	16 (10%)
Excluded: no usable channels in ROI	0 (0%)	2 (11%)	2 (15%)	3 (21%)	0 (0%)	4 (17%)	4 (15%)	7 (23%)	22 (14%)

### Stimuli

Stimuli consisted of trisyllabic sequences presented in either an ABB (e.g., “ba-lo-lo”) or ABC (e.g., “ba-lo-ti”) artificial grammar (see Gervain et al., 2008, for further details). Trisyllabic sequences were grouped into blocks of 10 sounds with a random inter-trial interval of 500–1500 ms. Each block lasted ~16 s and was separated by a silent pause of varying duration (15 s minimum). In general, the examiner initiated subsequent blocks after the 15 s silent pause that followed each block; however, in instances in which the infant became upset the experimenter would initiate the subsequent block only after the infant was no longer fussy. Up to 28 blocks were presented in one of two semi-randomized sequences.

### Procedure

**Figure 1:**
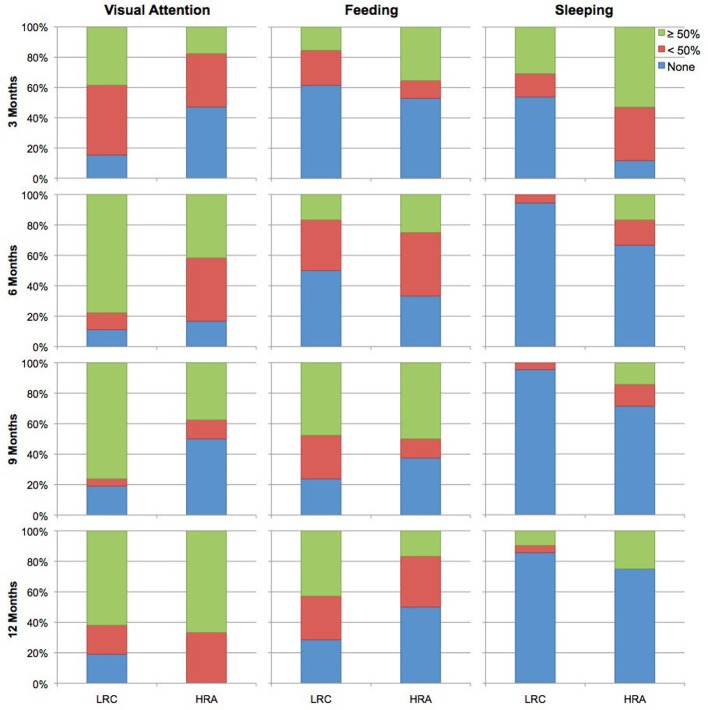
**Coded behavioral data for time spent attending to visual stimuli, feeding, and sleeping for LRC and HRA groups at 3-, 6-, 9-, and 12-months**.

Data were acquired in a dimly lit electrically- and acoustically-shielded room. Infants were seated on their caregivers' lap. During the visit, infants completed three tasks in the following order: (1) a NIRS experiment examining facial identity and emotion processing, (2) an upright-inverted face eye-tracking paradigm, and (3) the task reported here, a NIRS language processing paradigm. For this task, each block was initiated by an examiner who monitored the infant's movement. Blocks were presented until a total of 28 were completed or until the infant no longer tolerated the task. Infants were also presented with a continuous video of different moving shapes. If infants became uninterested in the video or upset, an experimenter used silent toys and bubbles in an attempt to keep the infant calm and still. Infants who became fussy were permitted to nurse, feed from a bottle, or to eat in order to expose them to as many blocks as possible. While these techniques have the potential to introduce motion artifacts, prior electrophysiological studies (a methodology more susceptible to motion artifacts than NIRS) have demonstrated sufficient amounts of artifact-free data can be acquired under similar circumstances. A subset of infants fell asleep during the course of the experiment; in these cases, the experiment proceeded as described above as most infant fcNIRS studies have been completed during natural sleep. Given the nature of the study visit (which required awake infants to attend to visual stimuli prior to the current experiment) and infant experimental research in general, infant state varied across the task (i.e., including awake and attending to visual information, eating or nursing, and/or asleep). Because connectivity measures are dependent on levels of wakefulness and arousal (e.g., awake vs. asleep; see Heine et al., 2012, for review) as well as task-related activation (e.g., Arfanakis et al., 2000), we examined whether groups differed with respect to the frequency of attentive, feeding, and sleeping states across the task. Based on notes taken from each visit, infant state was coded according to three broadly defined categories: (1) visual attention: infant watched video, bubbles, and/or silent toys, (2) feeding: infant nursed, fed from a bottle, or ate, and (3) sleep: infant fell asleep. Relative to the total number of blocks completed, infants were coded as whether they spent 0%, <50%, or ≥50% of their time in each state. Distributions of visual attention, feeding, and sleeping between groups were compared at each age using chi-squared tests (see Figure 1). Groups only differed significantly in the distribution of sleep at 3-months, *X*^2^(2, *n* = 30) = 6.3, *p* < 0.05, with a greater percentage of HRA infants sleeping relative to LRC infants. Furthermore, to confirm that variability of NIRS signal did not differ between groups, the root mean square (RMS) of the average ROI time courses was calculated (Larson-Prior et al., 2009). RMS did not differ between groups for any ROI at any time point with the exception of the left anterior ROI at 6-months, *t*_(28)_ = 2.1, *p* < 0.05, where the LRC group had significantly larger RMS compared to the HRA group.

### Near-infrared spectroscopy (NIRS)

#### Acquisition and processing

**Figure 2:**
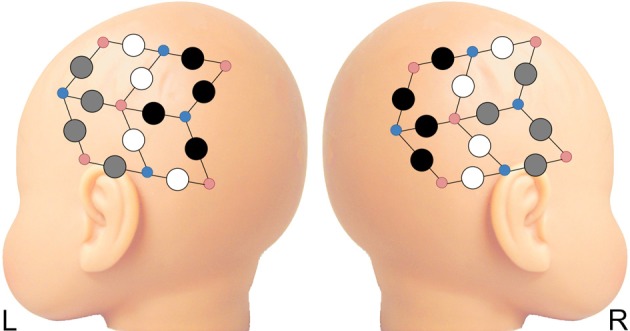
**Four regions of interest (ROI) selected from anterior and posterior recording sites on each hemisphere.** Gray circles represent channels included in anterior ROIs; black circles are channels included in posterior ROIs. Probes not included in ROIs are depicted in white. Smaller red and blue circles represent infrared emitters and detectors, respectively.

A 24-channel Hitachi ETG-4000 NIRS system was used to measure levels of oxy- and deoxy-hemoglobin (oxy-Hb and deoxy-Hb). Two wavelengths of light (695 and 830 nm) were used to detect hemodynamic responses with a sampling rate of 10 Hz. The NIRS probes were arranged in two 3 × 3 chevron arrays, each with five incident and four detecting fibers with 3 cm spacing. Each pair of emitting-detecting fibers defines a single channel. Probes were attached to a soft hat designed for infants (see Figure 2). NIRS probe sets were upgraded over the course of our longitudinal study. There was no significant difference between groups for the number of data sets collected with each probe set at 3-, 9-, or 12-months (*p* > 0.4); at 6-months, groups did differ on the distribution of data collected with old (HRA *n* = 9; LRC *n* = 22) vs. new (HRA *n* = 5; LRC *n* = 0) probes (*p* < 0.05). However, at the ages at which significant group differences emerged (i.e., 3- and 12-months), there were no significant main effects of probe type (new, old) or interactions between probe and group (*p* > 0.3) for overall mean connectivity.

Analyses were conducted on NIRS data that were acquired continuously 5 s prior to the onset of the first block until 10 s after the end of the final block. Average time series duration was approximately 15 min and did not differ between HRA and LRC groups at any age (*ps* > 0.3). Based on light intensity detection through each channel, relative concentrations of oxy-Hb and deoxy-Hb were calculated for the absorbance of each wavelength using the modified Beer-Lambert law. Data were then band-pass filtered (0.008 < f < 0.08) and the linear trend was removed. Next, given the impact of head motion on functional connectivity measured using fcMRI (Power et al., 2012; Van Dijk et al., 2012), a series of quality control procedures were conducted to insure that only artifact-free data were included in the functional connectivity analysis. First, individual time points were censored if the raw signal exceeded 4.95 (indicating saturation signals) or if total-Hb change exceeded 0.3 mM^*^mm within a two sample time window. Next, for each channel, the RMS of the first temporal derivative was calculated for the oxy-Hb signal; channels were excluded if the RMS exceeded a threshold of 0.25 or if more than 50% of time points exceeded saturation threshold.

#### Functional connectivity analysis

Similar to previous studies investigating functional connectivity in infants using NIRS (Homae et al., 2010, 2011), we chose to focus on oxy-Hb, as the oxy-Hb signal has a higher signal-to-noise ratio than deoxy-Hb (Tong and Frederick, 2010) and overlaps to a greater degree with functional networks defined by the fMRI BOLD signal (Duan et al., 2012). Four regions of interest (ROI) were selected from anterior and posterior locations for each hemisphere (LA, left anterior; LP, left posterior; RA, right anterior; RP, right posterior) (see Figure 2). The average time course for each ROI was calculated from signals from usable channels within each ROI. Because findings of over- and under-connectivity in ASD-related studies may be associated with specific methodological choices (Muller et al., 2011), we chose to examine the data using two separate pipelines–with and without task regression. For the task-regression pipeline (referred to below as intrinsic connectivity), task-related signal fluctuations were removed in order to examine intrinsic cortical connectivity. Task regressors for both ABB and ABC conditions were included in a general linear model to remove hemodynamic responses associated with auditory stimuli. Next, correlations between the residual time courses for each ROI pair (for all 6 ROI pairs) were computed. For the non-task-regressed pipeline (referred to below as co-activation connectivity), task related activation was not removed. Instead, correlations between mean time courses for each ROI pair were computed. For both pipelines, ROI pair correlations were transformed using Fisher's *r* to *z'* transformation. Next, mean *z'* scores were created for all (all 6 ROI pairs), inter-hemispheric (LA-RA, LA-RP, LP-RA, LP-RP; which includes both homo- and hetero-topic connections), and intra-hemispheric (LA-LP, RA-RP) ROI pairs. Finally, differences in functional connectivity were examined in a cross-sectional manner at 3-, 6-, 9-, and 12-months. *Z*-transformed data were entered into a series of independent-samples *t*-tests to assess between-group differences in connectivity at each time point. A secondary bootstrap analysis (10,000 iterations) was used to confirm *t*-test results. Shapiro–Wilk test of normality confirmed the data for each group met the normality assumption for tests that showed between-group differences. All statistical analyses were performed using SPSS, version 18.0.0.

## Results

**Figure 3:**
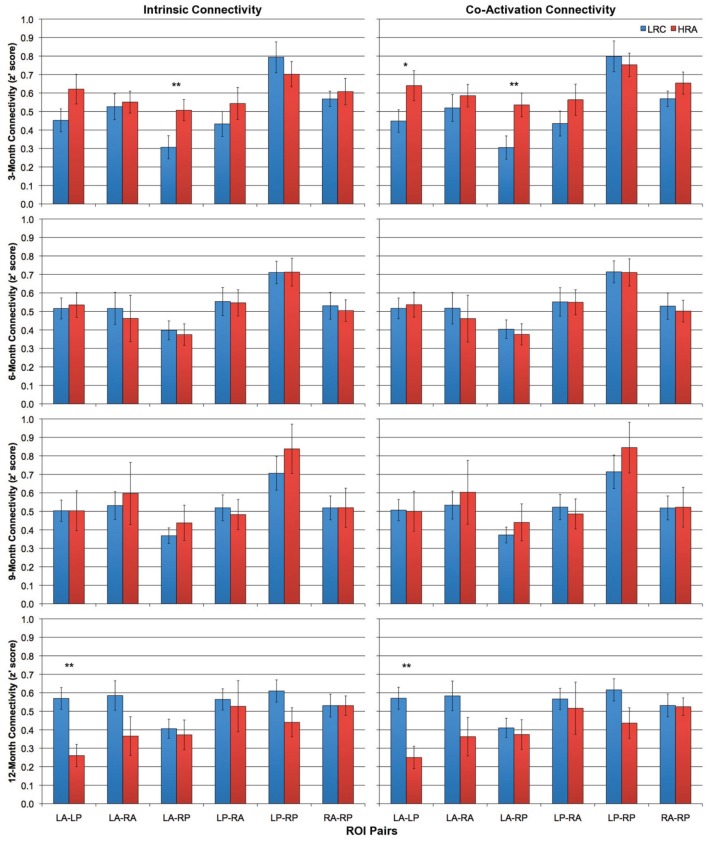
**Intrinsic (left column) and co-activation (right column) connectivity for HRA and LRC groups at 3-, 6-, 9-, and 12-months for all six ROI pairs.** Error bars represent one standard error of the mean. ^*^p < 0.1, ^**^p < 0.05.

Intrinsic and co-activation connectivity z-scores for all ROI pairs for both HRA and LRC groups at 3-, 6-, 9-, and 12-months are shown in Figure 3. At 3-months, differences between HRA and LRC infants for intrinsic connectivity were present only for the LA-RP ROI pair, *t*_(28)_ = −2.3, *p* < 0.05. More robust group differences emerged for co-activation connectivity as HRA infants showed marginally increased overall functional connectivity, *t*_(28)_ = −2.0, *p* = 0.054. This was mainly due to increased connectivity for intra-hemispheric ROI pairs, *t*_(28)_ = −2.3, *p* < 0.05. Analysis of individual ROI pairs revealed significantly increased connectivity between LA-RP, *t*_(28)_ = −2.5, *p* < 0.05, and marginally increased connectivity between LA-LP, *t*_(28) = −1.8_, *p* < 0.1, in HRA as compared to LRC infants. Results for both intrinsic and co-activation *t*-tests at 3-months were confirmed by a bootstrap analysis (10,000 iterations).

**Figure 4:**
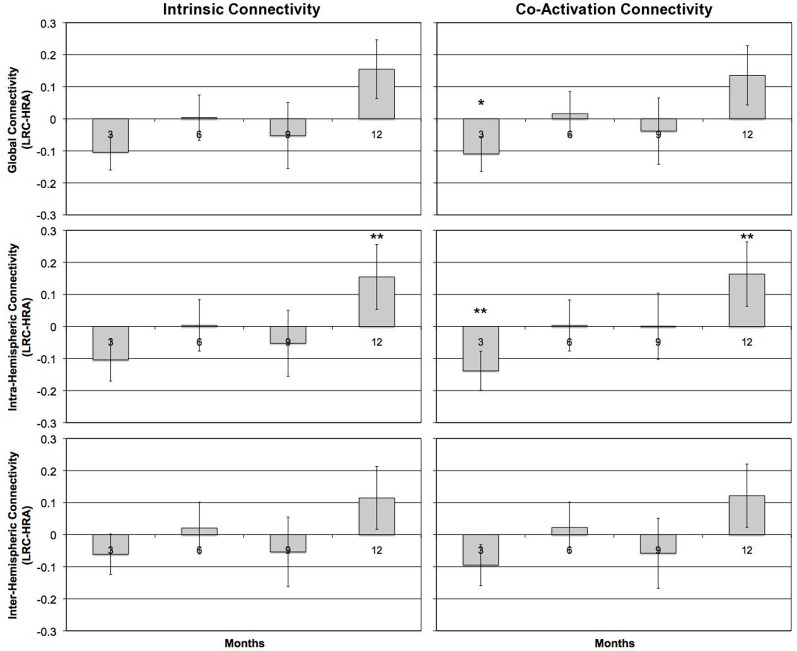
**Group differences in mean *z*'-scores for global connectivity and intra- and inter-hemispheric connectivity measures at 3-, 6-, 9-, and 12-months for task-regressed, intrinsic functional (left column) and co-activation (right column) connectivity pipelines.** Positive scores reflect LRC > HRA; negative scores reflect HRA > LRC for connectivity measures. Error bars represent one standard error of the mean. ^*^p < 0.1, ^**^p < 0.05.

There were no significant differences between any average *z*' score or individual ROI pair for either intrinsic or co-activation analysis at 6- or 9-months (all *p* > 0.4). However, by 12-months, LRC infants showed increased intrinsic connectivity relative to HRA infants. Specifically, LRC infants had increased intra-hemispheric connectivity relative to HRA infants, *t*_(20.7)_ = 2.3, *p* < 0.05, which was primarily due to significantly increased connectivity of the LA-LP ROI pair, *t*_(25)_ = 2.7, *p* < 0.05. Increases in global connectivity did not reach significance; however, as can be seen in Figure 4, differences in connectivity across the first year of life shift from marginally increased connectivity for HRA infants at 3-months to increased connectivity for LRC infants by 12-months. Findings for co-activation connectivity were identical to intrinsic connectivity results; relative to the LRC group, the HRA group showed decreased connectivity for intra-hemispheric connections, *t*_(22.2)_ = 2.5, *p* < 0.05, which was driven by significantly decreased LA-LP connectivity, *t*_(25)_ = 2.7, *p* < 0.05. Results for both intrinsic and co-activation *t*-tests at 12-months were confirmed by a bootstrap analysis (10,000 iterations) with the exception of mean intra-hemispheric connectivity, which was marginally increased in LRC infants for co-activation analysis, *t*_(25)_ = 1.6, *p* < 0.1, and no longer significant for intrinsic analysis, *t*_(25)_ = 1.5, *p* > 0.1.

## Discussion

The current study is the first to use NIRS to examine functional connectivity in infants at-risk for developing ASD. Our preliminary findings suggest that divergent patterns of functional connectivity emerge across the first year of life. Whereas LRC infants showed a pattern of increasing functional connectivity from 3- to 12-months, HRA infants exhibited a pattern of *decreasing* connectivity. These contrasting patterns resulted in increased connectivity at 3-months in HRA compared to LRC infants and, by 12-months, decreased connectivity in HRA compared to LRC infants.

Differences in functional connectivity at 3-months suggest that prenatal or early postnatal differences in brain connectivity exist in infants at-risk for ASD. A comparison to the findings of Wolff et al. (2012), which previously reported early connectivity differences in at-risk infants, is difficult because the study did not include a neurotypical comparison group. However, findings from other DTI studies suggest elevated indices of white matter connectivity and, perhaps, accelerated white matter growth (Ben Bashat et al., 2007; Weinstein et al., 2011), which is followed by reduced FA in school-aged children, adolescents, and adults with ASD (Travers et al., 2012). Our findings of increased functional connectivity at 3-months is in agreement with the idea that, early in development, individuals with (or at-risk for) ASD may potentially have diffusely increased connectivity.

However, similar to Dinstein et al. (2011) fcMRI study of toddlers with ASD, our preliminary 12-month results show reduced connectivity of both anterior and posterior inter-hemispheric connections (albeit not significantly so). Further, our results show that, at 12-months, HRA infants have reduced intra-hemispheric connectivity (both co-activation and intrinsic) for the left hemisphere compared to LRC infants. These results, in conjunction with weaker inter-hemisphere connectivity of inferior frontal and superior temporal gyri reported by Dinstein et al. (2011), suggest that early atypical development of the language-processing network may exist in infants and toddlers at risk for or diagnosed with ASD. Although we are currently unable to determine whether differences in connectivity at 3- and 12-months were driven by infants that will later go on to meet diagnostic criteria for ASD, the results add to a growing body of evidence suggesting that atypical connectivity may be an potential endophenotype for ASD (e.g., Barnea-Goraly et al., 2010).

Previous functional connectivity studies in neurotypical adults have shown state (e.g., awake vs. asleep) may alter degree of network connectivity (see Heine et al., 2012, for review). Although state-dependent deviations in connectivity may be network-specific and vary according to level of wakefulness [e.g., descent to sleep (Larson-Prior et al., 2009) vs. deep sleep (Horovitz et al., 2009)], reduced levels of awareness are generally associated with decreased levels of connectivity. In the current study, NIRS data were acquired during different levels of wakefulness and arousal. Differences in the distribution of visual attention, feeding, and sleeping were similar for HRA and LRC infants except at 3-months where a larger proportion of at-risk infants slept during the task compared to the LRC infants. Assuming similar properties of connectivity dynamics exist in the infant brain (which remains undetermined as no study to date has examined differences in functional connectivity in sleep-wake states in infants), we would assume that high-risk infants would show reduced connectivity relative to LRC infants based on state alone. However, our results show that infants at-risk have increased connectivity relative to low-risk infants despite spending a larger portion of the assessment sleeping.

Although infants at risk for ASD have been shown to have similar brain volume measurements at 6-months compared to LRC infants (Hazlett et al., 2012; Shen et al., 2013), ASD is associated with accelerated brain growth over the first years of life (Redcay and Courchesne, 2005; Shen et al., 2013). Lewis and Elman (2008) hypothesized that early overgrowth results in atypical patterns of connectivity, specifically reduced long-distance connectivity, and demonstrated that developmental differences in connectivity emerged at between 12 and 24 simulated months using a neural network model. Further, Lewis et al. (2009) have shown that larger brains are associated with reduced long distance connectivity (potentially due to increased conduction delays and cellular costs associated with long-distance connections), and that corpus callosum size in individuals with ASD is inversely related to intracranial volume (i.e., larger brain, smaller corpus callosum) (Lewis et al., 2012). Although the current study did not find any between-group differences in head circumference, future studies may wish to examine the relations between trajectories of brain size or head circumference and the emergence of group differences in patterns of anatomical and functional connectivity.

Lastly, the current study employed task-regressed, intrinsic and non-task-regressed, co-activation analyses as task regression in fcMRI studies may result in different patterns of over- and under-connectivity in ASD (Jones et al., 2010; Muller et al., 2011). Although general patterns of over- and under-connectivity were consistent for both methods across 3-, 6-, 9-, and 12-month time points, group differences (specifically, increased connectivity in the HRA group) were more robust for co-activation analyses at 3-month of age.

### Limitations

There are several limitations to the current study. First, our sample sizes, especially for the 9- and 12-month time points, are small and therefore the current results should be viewed as preliminary and interpreted with caution. Furthermore, small sample sizes restricted current analyses to a cross-sectional examination of the data. Future studies with larger sample sizes will employ longitudinal statistical analyses to examine developmental trajectories of functional connectivity across the first year of life. Second, measurement of oxy- and deoxy-Hb responses requires transmission of light through scalp, skull, cerebral spinal fluid, and meninges; however, scalp-brain distance increases across development and is significantly shorter in the left compared to right hemisphere (Beauchamp et al., 2011). Additionally, the presence of hair (which increases throughout development) can result in the attenuation of light and result in unreliable measurements. It is unclear how these developmental changes differentially impact low- and high-risk infants (although see Shen et al., 2013, for example of differences in cerebral spinal fluid); nevertheless, future studies may wish to address these potentially confounding issues. Third, although levels of wakefulness and arousal varied within each infant's visit, the distribution of infant state rarely varied across group. Nevertheless, the current results should be interpreted with caution as subtle variations in infant state could have potentially impacted our group comparisons. Additionally, while we took steps to remove time points and channels corrupted by movement artifacts, head motion was not measured in the current study and therefore we are unable to determine whether group differences in motion artifacts were present. Lastly, ROIs in the current study included large areas of lateral frontal and posterior cortex and are therefore unlikely to sample homogeneous cortical areas. As a result, our current measure has limited spatial resolution, which is likely to introduce variability within our connectivity measures.

## Conclusions

Distributed functional brain networks arise from the complex interaction of genes, environmental factors, and experience-dependent processes. Our findings suggest that, in infants with a family history of ASD, there are early differences in brain connectivity and an atypical developmental trajectory of functional connectivity compared to LRC infants. Because the majority of our current sample of infants have yet to reach 36 months of age, we do not have data regarding diagnostic outcome. Therefore, our current analysis has only examined whether *risk* for autism is associated with differences in connectivity (i.e., an endophenotype), rather than whether infants that are later diagnosed with ASD exhibit unique patterns of connectivity in the first year of life. In conjunction with previous findings (e.g., Barnea-Goraly et al., 2010), the current results suggest that atypical network connectivity may represent a putative endophenotype in ASD. Although our findings are in accord with other functional and structural connectivity studies of high-risk infants and toddlers with ASD (Dinstein et al., 2011; Wolff et al., 2012), our results should be interpreted with caution given the small sample sizes. Ongoing data collection will provide a larger sample for more sophisticated longitudinal analyses, as well as the ability to examine whether differences in connectivity exist between high-risk infants that do and do not go on to develop ASD.

### Conflict of interest statement

The authors declare that the research was conducted in the absence of any commercial or financial relationships that could be construed as a potential conflict of interest.
